# Spider fauna (Arachnida, Araneae) in Mordovia State Nature Reserve and National Park “Smolny” (Russia)

**DOI:** 10.3897/BDJ.11.e105979

**Published:** 2023-10-20

**Authors:** Sergei Esyunin, Oksana Agafonova, Alexander Ruchin, Gennadiy Semishin, Mikhail Esin, Oleg Artaev

**Affiliations:** 1 Perm State University, Perm, Russia Perm State University Perm Russia; 2 Joint Directorate of the Mordovia State Nature Reserve and National Park «Smolny», Saransk, Russia Joint Directorate of the Mordovia State Nature Reserve and National Park «Smolny» Saransk Russia; 3 Papanin Institute for Biology of Inland Waters Russian Academy of Sciences, Borok, Russia Papanin Institute for Biology of Inland Waters Russian Academy of Sciences Borok Russia

**Keywords:** species diversity, protected areas, species conservation, dataset, European Russia

## Abstract

**Background:**

Ecosystems in protected areas are richer in animal and plant species diversity ("biodiversity hotspots") due to more stringent conservation conditions. Of particular importance is scientific research and monitoring of this diversity in such areas. The aim of this study is to describe a set of data on Araneae occurrence in two protected areas: Mordovia State Nature Reserve and National Park "Smolny".

**New information:**

About 29,000 individuals are identified to the species level. In total, 342 species were recorded for both PAs. The greatest species diversity was recorded in the families Linyphiidae (109 species; 32%), Lycosidae (38 species; 11%) Gnaphosidae (28 species; 8%), Araneidae (25 species; 7%), Salticidae (24 species; 7%), Thomisidae (23 species; 7%) and Theridiidae (22 species; 6% from total species diversity). The five species most abundant in the lower stratum (litter and moss layer) of biocenoses were *Diplocephaluspicinus*, *Micronetaviaria*, *Tenuiphantestenebricola*, *Diplostylaconcolor* and *Abacoproecessaltuum* and the five species most abundant in the vegetative stratum (herb, shrub and tree stems and canopy) were *Linyphiatriangularis*, *Enoplognathaovata*, *Evarchafalcata*, *Misumenavatia* and *Evarchaarcuata*. The dataset contains information on the occurrence of seven rare species (*Centromerusnurgush*, *Centromeruspersimilis*, *Diplocephalusdentatus*, *Entelecaraflavipes*, *Metapanamomopskaestneri*, *Pelecopsisradicicola* and *Porrhommamicrocavense*), three species (*Agalenatearedii*, *Neosconaadianta*, *Thanatusoblongiusculus*) that entered here from the steppe zone and two synanthropic species (*Steatodacastanea*, *Tegenariadomestica*).

## Introduction

Playing an important role in addressing current challenges, protected areas have been a key conservation strategy for many years. The establishment, sustainable operation, permanent protection and large-scale study of protected areas is a central strategy for global biodiversity conservation (Geldmann et al. 2013, Graham et al. 2021). Formal establishment of protected areas is one of the most important conservation actions to mitigate these losses. Globally, the coverage of terrestrial protected areas has increased from 14.1% to 15.3% in the last decade and this trend is expected to continue in line with international political commitments to conservation (Maxwell et al. 2020), the most significant of which - "30 by 30" - is a worldwide initiative for governments to designate 30% of Earth's land and ocean area as protected areas by 2030 (Dinerstein et al. 2019).

Of particular importance to protected areas is the study of their biodiversity (Minin et al. 2020, Wangmo et al. 2021, Shashkov et al. 2022). A key challenge for understanding current drivers of biodiversity loss and conservation effectiveness is the relatively slow rate of biodiversity assessment. Many studies in Eurasia, in particular, in Russia, have shown that the number of species in protected areas is much higher than in neighbouring areas that humans actively use for their activities (Gray et al. 2016, Dedyukhin 2022, Kaicheen and Mohd-Azlan 2022).

Spiders (Arachnida, Araneae) are found almost all over the Planet, they have colonised all natural areas, except the open sea and air. They are amongst the most abundant arthropods in most terrestrial habitats, where they play an important ecological role (Plath et al. 2021, Van Klink et al. 2021, Milano et al. 2021). Spiders are the largest taxonomic group that consists entirely of predators. Part of their successful distribution and species diversity can be explained due to their ability to colonise almost all terrestrial habitats (Laborda et al. 2018, Gajdoš et al. 2022, Narimanov et al. 2022). Spider assemblages are particularly diverse in areas rich in vegetation, but they can also be found in habitats with strict ecological constraints, such as deserts, caves and high-altitude habitats (Banaag et al. 2020, Kawalkar et al. 2022, Tanasevitch and Rybalov 2022). The total current spider biodiversity is about 51 thousand species (World Spider Catalogue, Anonymous 2023). As of 31 December 2017, 2445 spider species have been recorded in Russia (Mikhailov 2021).

The aim of this study is to describe a dataset on the occurrence of Araneae in two protected areas: Mordovia State Nature Reserve (MSNR) and National Park "Smolny" (NPS). The dataset was recently published in the Global Biodiversity Information Facility as the main Darwin Core archive (Esyunin et al. 2022). This is the first complete description of the Araneae fauna of forest ecosystems of protected areas, located in central European Russia.

## General description

### Additional information

A total of 341 spider species from 161 genera of 25 families were identified (Table 1). In MSNR, there are 318 species (152 genera, 25 families) and in NPS, there are 222 species (114 genera, 21 families).

The Linyphiidae has the greatest taxonomic diversity in the general collection, constituting almost one third (32%) of species and 40% of genera. Six other spider families have approximately equal, high species diversity (Table 1): Lycosidae (11%), Gnaphosidae (8%), Araneidae, Salticidae and Thomisidae (7% each), Theridiidae (6% of total species diversity).

In MSNR and NPS spider collections, the ratios of families are not fundamentally different. As in the general collection, the family Linyphiidae has the highest species and genus diversity (Table 1). Linyphiidae include 41% and 31% of genera and 31% and 24% of species found in MSNR and NPS, respectively. Lycosidae are the second in terms of species diversity in both territories, 12% and 14% of spider species belong to them in MSNR and NPS, respectively. Three spider families, namely Araneidae, Gnaphosidae and Thomisidae (7-9% of total species) have high diversity in both territories. In addition, the family Theridiidae (6% of the total number of species) is diverse in MSNR.

The studied faunas belong to the polytaxon-liniphyid type - faunas of this type are characterised by predominance of spiders of family Linyphiidae (Esyunin 2015a), which make up about half of the species diversity and there are at least three families with an abundance share of at least 5%. There is also a significant "plume" of families with high species diversity, which are characteristic of the southern taiga subzone of the boreal zone and subboreal zone of the Russian Plain (Esyunin and Efimik 1994, Esyunin et al. 2011).

Most of the captured individuals belong to Lycosidae (62% of the total number of individuals). Gnaphosidae (12%), Thomisidae (7%) and Linyphiidae (7% of the total number of individuals) are abundant in the total collection; Araneidae, Tetragnathidae and Theridiidae are relatively numerous (about 2-3% of the total number of individuals). The families Agelenidae and Theridiosomatidae are represented by single individuals (Table 1).

Spider species can be associated with one or more vegetation layers (Sozontov and Esyunin 2022), so it is rational to consider the most abundant species by the biocenosis stratum. Amongst species inhabiting the lower stratum (litter and moss layer) of the biocenosis, the most abundant in our collection of linyphiids are *Diplocephaluspicinus* (Blackwall, 1841), *Micronetaviaria* (Blackwall, 1841), *Tenuiphantestenebricola* (Wider, 1834), *Diplostylaconcolor* (Wider, 1834) and *Abacoproecessaltuum* (L. Koch, 1872) (Table 2). Species confined to vegetation (above-ground stratum; herb layer, shrub layer, tree stems and canopy layer) are relatively abundant in our collection. Amongst them, *Enoplognathaovata* (Clerck, 1757), *Evarchafalcata* (Clerck, 1757), *Evarchaarcuata* (Clerck, 1757), *Linyphiatriangularis* (Clerck, 1757) and *Misumenavatia* (Clerck, 1758) are the most numerous species (Table 2). Amongst the numerous epigean spiders (herpetobium strate; ground surface), the wolf-spiders *Alopecosaaculeata* (Clerck, 1757), *Pardosaalacris* (C. L. Koch, 1833), *Pardosalugubris* (Walckenaer, 1802), *Piratulahygrophyla* (Thorell, 1872), *Trochosaterricola* Thorell, 1856 and *Xerolycosanemoralis* (Westring, 1861) are the most abundant (Table 2).

Three steppe species are found in the NPS fauna: *Agalenatearedii* (Scopoli, 1763), *Neosconaadianta* (Walckenaer, 1802) and *Thanatusoblongiusculus* (Lucas, 1846), located at the northern border of their ranges within the East European Plain.

A number of rare spider species are present in the collection. For the species *Centromerusnurgush* Tanasevitch & Esyunin, 2013, recently described from the Kirov Region, in the MSNR is the second finding. The European species *Porrhommamicrocavense* Wunderlich, 1990 is known from several locations in Central and Eastern Europe (Růžička 2018), but in the East European Plain, it is known only from Perm Region (Esyunin 2007) and Mordovia. A similar situation is observed for European species, *Centromeruspersimilis* (O. Pickard-Cambridge, 1912), which are relatively widely distributed in Europe, but, in the East European Plain, are known from Mordovia and the Permian Urals (Nentwig et al. 2023). For three European species, *Diplocephalusdentatus* Tullgren, 1955, *Entelecaraflavipes* (Blackwall, 1834) and *Pelecopsisradicola* (L. Koch, 1872), the indications for Mordovia are the eastern range border.

The presence of permanent living quarters on the studied territory explains the presence in the fauna of two synanthropic species *Steatodacastanea* (Clerck, 1757) and the domestic house spider *Tegenariadomestica* (Clerck, 1757).

The number of species found in the MSNR and NPS faunas are 1.5-2.0 times less than the diversity of faunas located in the west of the East European Plain in the subboreal zone (Biebrza National Park and Belovezhskaya Pushcha National Park) or on the border with it (Nizhne-Svirskii State Nature Reserve) (Table 3). It is likely that these protected areas are examples of a territory with an increased species diversity of spiders (biodiversity hot point), an overview of which was given earlier (Marusik and Koponen 2002, Mikhailov and Trushina 2013).

## Sampling methods

### Study extent

We used standard collecting methods: pitfall-traps, sweeping, litter sifting and manual collecting (Oliger 2010, M. and Kovblyuk 2011, Golub et al. 2012). The main part of the material is obtained from soil traps with a fixator, collecting from herbaceous vegetation and shrubs, by shaking off bushes and low trees. Collection sites are typical biotopes of two protected areas - forests of various types, structures, origins and ages (pine forests, mixed forests, spruce forests, birch forests, broad-leaved forests, floodplain wet broad-leaved forests, alder forests), forest clearings and meadow stands. Preservation was carried out either in traps (soil traps with formalin) or alcohol when caught by other methods. The main volume of the material was collected by A.B. Ruchin, O.V. Agafonova, M.N. Yesin and G.B. Semishin. Part of the material on which the dataset was created was used in publications (Mikhailov and Trushina 2013, Ruchin et al. 2013, Esyunin et al. 2020, Esyunin et al. 2021).

## Geographic coverage

### Description

**Figure 1.**
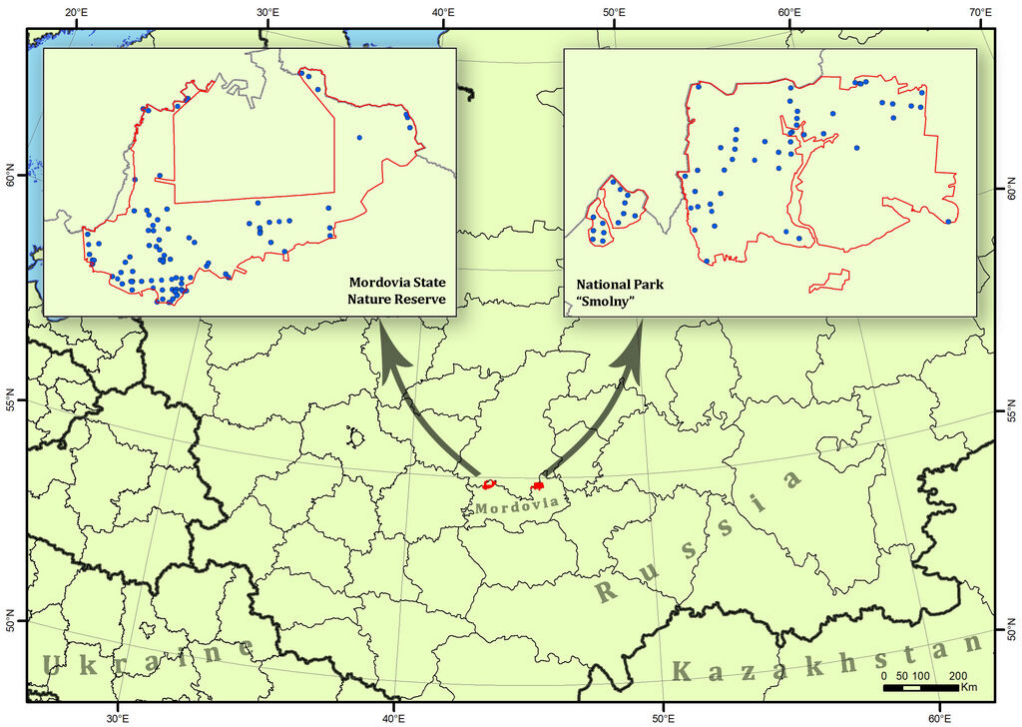
Map of the locations of the studied protected areas and occurrences (blue points) in them.

Both study areas are located in the centre of the Russian plain in the Republic of Mordovia (central European part of Russia) (Fig. 1). The MSNR is located in the Oka-Don Lowland. The MSNR covers 321.62 km^2^. Its boundary position at the juncture of two landscape zones, forest-steppe and forest, is an important distinguishing feature of the territory. The MSNR area itself is located in the broad-leaved forest zone, but its eastern, south-eastern and southern borders coincide with the border of the forest-steppe zone. Forest ecosystems occupy 89.3% of the area. Forests are mainly represented by pine and mixed forests. Birch and alder forests form on the areas burned in 2010 and 2021. Broadleaved forests are few and mostly located in the northern, western and south-western parts in the floodplains of the rivers. The MSNR is climatically part of the Atlantic-continental temperate zone. Since 1936 when the MSNR was established, some warming has been recorded, i.e. an increase in mean annual precipitation and a degree of continental climate (Ruchin et al. 2019, Tereshkina et al. 2020) have been observed.

The NPS is located in the Volga Upland. It covers an area of 363.86 km^2^. Pine forests are the main type of forest ecosystems. They are mainly located in the southern and central parts. The northern part is dominated by broad-leaved forests, where the main forest forming species are linden (*Tiliacordata* Mill.), oak (*Quercusrobur* L.), maple (*Acerplatanoides* L.), ash (*Fraxinusexcelsior* L.) and elm (*Ulmus* spp.). Alder groves form in the floodplains of some rivers and streams (Kirillov and Kirillova 2021). In contrast to the MSNR, there are many more open areas in the NPS. These are large glades, places where former settlements were located, clearings under power lines etc. The majority of NPS forest ecosystems are man-made, for example pine forests. Broad-leaved forests were most often formed on sites of large clearcuts before the NPS was established and originated from stumps on the site of clearcuts. The climate is moderately continental.

### Coordinates

54.7087 and 54.9309 Latitude; 45.6119 and 43.0750 Longitude.

## Taxonomic coverage

### Description

Species names are given according to the World Spider Catalogue (Anonymous 2023). All taxa are listed in alphabetic order; nomenclature follows the World Spider Catalogue (Anonymous 2023).

### Taxa included

**Table:** 

Rank	Scientific Name	Common Name
order	Araneae	Spiders

## Temporal coverage

**Formation period:** 2008; 2010-2013; 2015-2021.

### Notes

Materials include collections of 2008, 2010-2013, 2015-2021.

## Usage licence

### Usage licence

Creative Commons Public Domain Waiver (CC-Zero)

## Data resources

### Data package title

Spiders (Arachnida: Araneae) Mordovia State Nature Reserve and National Park "Smolny" (Russia)

### Resource link

https://www.gbif.org/dataset/fbada6af-3d46-4ef5-8c88-df92e037459f

### Alternative identifiers

https://doi.org/10.15468/mgmj3e

### Number of data sets

1

### Data set 1.

#### Data set name

Spiders (Arachnida: Araneae) Mordovia State Nature Reserve and National Park "Smolny" (Russia)

#### Data format

Darwin Core

#### Description

Dataset contains data on the occurrence of Araneae in two protected areas of Mordovia (Russia): Mordovia State Nature Reserve and National Park "Smolny". Research was carried out in 2008, 2010–2013 and 2015–2021. Data on Araneae species from 25 families are presented.

**Data set 1.** 

Column label	Column description
occurrenceID	An identifier for the occurrence (as opposed to a particular digital record of the occurrence).
basisOfRecord	The specific nature of the data record: HumanObservation
eventDate	The number of individuals represented present at the time of the occurrence.
scientificName	The full scientific name including the genus name and the lowest level of taxonomic rank with the authority.
kingdom	The full scientific name of the kingdom in which the taxon is classified.
phylum	The full scientific name of the phylum or division in which the taxon is classified.
class	The full scientific name of the class in which the taxon is classified.
order	The full scientific name of the order in which the taxon is classified.
family	The full scientific name of the family in which the taxon is classified.
taxonID	An identifier for the set of taxon information. Taxon details in World Spider Catalogue.
taxonRank	The taxonomic rank of the most specific name in the scientificName.
decimalLatitude	The geographic latitude of location in decimal degrees.
decimalLongitude	The geographic longitude of location in decimal degrees.
geodeticDatum	The ellipsoid, geodetic datum or spatial reference system (SRS) upon which the geographic coordinates given in decimalLatitude and decimalLongitude are based. Here - WGS84.
coordinateUncertaintyInMeters	The horizontal distance (in metres) from the given decimalLatitude and decimalLongitude describing the smallest circle containing the whole of the Location.
locality	The specific description of the place.
Country	The name of the country in which the Location occurs. Here - Russia.
countryCode	The standard code for the country in which the Location occurs. Here - RU.
individualCount	The number of individuals represented present at the time of the occurrence.
year	The integer day of the month on which the Event occurred.
month	The ordinal month in which the Event occurred.
day	The integer day of the month on which the Event occurred.
recordedBy	A person, group or organisation responsible for recording the original occurrence.
identifiedBy	A list of names of people, who assigned the Taxon to the subject.

## Figures and Tables

**Table 1.** Species diversity of Araneae families from the dataset

Family	Number of individuals	Total of species	Number of genera		Number of species	
			MSNR	NPS	MSNR	NPS
Agelenidae	3	2	2	0	2	0
Anyphaenidae	14	1	1	0	1	0
Araneidae	470	25	11	12	22	21
Asteraceae	38	1	1	1	1	1
Cheiracanthidae	15	5	1	1	3	3
Clubionidae	141	10	1	1	10	6
Dictynidae	33	6	3	1	6	2
Gnaphosidae	3604	28	6	6	27	21
Hahniidae	45	5	3	3	5	4
Linyphiidae	1922	109	62	35	100	54
Liocranidae	663	5	2	2	5	5
Lycosidae	17909	38	10	9	38	31
Mimetidae	12	2	1	1	1	2
Miturgidae	115	2	1	1	2	2
Oxyopidae	12	1	1	1	1	1
Philodromidae	201	13	3	3	11	9
Phrurolithidae	29	1	1	1	1	1
Pisauridae	65	3	2	2	3	3
Salticidae	410	24	13	12	22	15
Sparassidae	36	1	1	1	1	1
Tetragnathidae	747	13	3	3	12	10
Theridiidae	460	22	14	10	21	11
Theridiosomatidae	1	1	1	0	1	0
Thomisidae	1932	23	7	8	21	19
Titanoecidae	38	1	1	0	1	0
**Total**	**28915**	**342**	**152**	**114**	**318**	**222**

**Table 2.** Number of specimens of more abundant spider species of Araneae from the dataset.

Species	Number of specimens
Low stratum (litter and moss layer)	
*Diplocephaluspicinus* (Blackwall, 1841)	189
*Micronetaviaria* (Blackwall, 1841)	147
*Tenuiphantestenebricola* (Wider, 1834)	129
*Diplostylaconcolor* (Wider, 1834)	76
*Abacoproecessaltuum* (L. Koch, 1872)	65
Vegetation stratum (herb layer, shrub layer, tree stems and canopy layer)	
*Linyphiatriangularis* (Clerck, 1757)	214
*Enoplognathaovata* (Clerck, 1757)	143
*Evarchafalcata* (Clerck, 1757)	110
*Misumenavatia* (Clerck, 1758)	110
*Evarchaarcuata* (Clerck, 1757)	102
*Xysticusulmi* (Hahn, 1831)	92
*Tibellusoblongus* (Walckenaer, 1802)	88
*Nerieneradiata* (Walckenaer, 1841)	82
*Ebrechtellatricuspidata* (Fabricius, 1775)	78
*Linyphiahortensis* Sundevall, 1830	77
*Metellinasegmentata* (Clerck, 1757)	76
*Helophorainsignis* (Blackwall, 1841)	74
*Clubionacaerulescens* L. Koch, 1867	66
*Singahamata* (Clerck, 1757)	67
*Mangoraacalypha* (Walckenaer, 1802)	54
Herpetobium stratum (ground surface)	
*Pardosalugubris* (Walckenaer, 1802)	4490
*Trochosaterricola* Thorell, 1856	4000
*Piratulahygrophyla* (Thorell, 1872)	3844
*Pardosaalacris* (C.L. Koch, 1833)	1568
*Xerolycosanemoralis* (Westring, 1861)	1172
*Alopecosaaculeata* (Clerck, 1757)	1028
*Zelotessubterraneus* (C.L. Koch, 1833)	679
*Ozyptilapraticola* (C.L. Koch, 1837)	598
*Xysticusluctator* L. Koch, 1870	633
*Haplodrassussoerenseni* (Strand, 1900)	610
*Agroecabrunnea* (Blackwall, 1833)	552
*Pachygnathalisteri* Sundevall, 1830	498
*Gnaphosabicolor* (Hahn, 1833)	441
*Haplodrassussilvestris* (Blackwall, 1833)	392
*Alopecosapulverulenta* (Clerck, 1757)	365
*Haplodrassusumbratilis* (L. Koch, 1866)	285
*Haplodrassussignifer* (C.L. Koch, 1839)	275
*Zelotesclivicola* (L. Koch, 1870)	244
*Xerolycosaminiata* (C.L. Koch, 1834)	229
*Alopecosacuneata* (Clerck, 1757)	201
*Trochosaruricola* (De Geer, 1778)	169
*Xysticusluctuosus* (Blackwall, 1836)	145
*Drassylluspraeficus* (L. Koch, 1866)	137
*Pardosaprativaga* (L. Koch, 1870)	121
*Pardosafulvipes* (Collett, 1876)	110
*Euryopisflavomaculata* (C.L. Koch, 1836)	103
*Robertuslividus* (Blackwall, 1836)	92
*Zelotesazsheganovae* Esyunin et Efimik, 1992	99
*Drassylluspusillus* (C.L. Koch, 1833)	89
*Zoraspinimana* (Sundevall, 1833)	71
*Zelotespetrensis* (C.L. Koch, 1839)	55
*Alopecosasulzeri* (Pavesi, 1873)	61
*Liocranoecastriata* (Kulczyński, 1882)	48
*Piratapiraticus* (Clerck, 1757)	49

**Table 3.** Comparison of Araneae fauna richness of different protected areas within East European Plain.

**Protected areas**	**Number of species**	**References**
Mordovia State Nature Reserve	318	Our data
National Park «Smolny»	222	Our data
Biebrza National Park	481	Kupryjanowicz (2005)
Belovezhskaya Pushcha National Park	437	Zhukovets (2017)
Kivach State Nature Reserve	272	Tselarius (1980)
Nizhne-Svirskii State Nature Reserve	434	Oliger (2016)
«Preduralie» Reserve	332	Esyunin et al. (2011)
Bashkiriya State Nature Reserve	276	Esyunin (2015b)
